# The impact of meropenem shortage and post-prescription review and feedback on broad-spectrum antimicrobial use: An interrupted time-series analysis

**DOI:** 10.1016/j.infpip.2024.100380

**Published:** 2024-06-22

**Authors:** Kohei Maruyama, Kiyoshi Sekiya, Noriyuki Yanagida, Shuhei Yasuda, Daisuke Fukumoto, Satoshi Hosoya, Hiromitsu Moriya, Motoko Kawabe, Tatsuya Mori

**Affiliations:** a Division of Antimicrobial Stewardship Program, NHO Sagamihara National Hospital, Sagamihara, Kanagawa, Japan; b Department of Pharmacy, NHO Sagamihara National Hospital, Sagamihara, Kanagawa, Japan; c Department of Allergy and Respirology, NHO Sagamihara National Hospital, Sagamihara, Kanagawa, Japan; d Department of Pediatrics, NHO Sagamihara National Hospital, Sagamihara, Kanagawa, Japan; e Department of Clinical Laboratory, NHO Sagamihara National Hospital, Sagamihara, Kanagawa, Japan; f Department of Nursing, NHO Sagamihara National Hospital, Sagamihara, Kanagawa, Japan; g Department of Emergency, Critical Care Medicine, NHO Sagamihara National Hospital, Sagamihara, Kanagawa, Japan; h Department of Surgery, NHO Sagamihara National Hospital, Sagamihara, Kanagawa, Japan

**Keywords:** Meropenem, Shortage, Post-prescription review and feedback

## Abstract

**Background:**

Meropenem (MEPM) holds significance in treating severe infections and drug-resistant bacteria. There are concerns that antimicrobial shortages may lead to the use of alternative antimicrobials that are less effective and safer. We have responded to the MEPM shortage with post-prescription monitoring and feedback (PPRF) with no restrictions on MEPM initiation. We aimed to assess the impact of the MEPM shortage and the PPRF on broad-spectrum antimicrobial use and mortality.

**Methods:**

This retrospective study was conducted in a single hospital in Japan. The period from October 2021 to August 2022 was defined as the period before the MEPM shortage, and the period from September 2022 to March 2023 was defined as the period during the MEPM shortage. To support the appropriate use of antimicrobials during MEPM shortages, the antimicrobial stewardship team (AST) developed a list of alternatives to MEPM. An interrupted time series analysis was used to assess changes in use and mortality among patients receiving broad-spectrum antimicrobials over the study period.

**Discussion:**

The shortage of MEPM and PPRF temporarily increased the use of alternative cefepime; however, the subsequent change in days of therapy and days of coverage of broad-spectrum antimicrobials suggests a decrease in the use of these antimicrobials. Despite these shifts, the mortality rates remained stable, suggesting that the response to the shortage did not adversely affect treatment outcomes.

**Conclusion:**

In the context of antimicrobial shortages, AST support plays an important role in enabling physicians to make optimal use of antimicrobials.

## Introduction

Meropenem (MEPM) is an important antimicrobial for the treatment of severe infections and drug-resistant bacteria [1]. However, the increasing resistance of Enterobacterales and *Acinetobacter baumanni*i to carbapenems is a global problem [2]. Therefore, unnecessary administration of carbapenems should be avoided to reduce the development of these drug-resistant organisms. Furthermore, conservation of carbapenems should be considered when there is an evidence-based alternative that ensures efficacy and safety.

Recently, several antimicrobials have faced supply shortages due to manufacturing issues, a common concern in Japan and globally [3,4]. In a 2018 survey of drug shortages in Europe, antimicrobial shortages were the most commonly reported [5]. The antimicrobial shortage may lead to the widespread use of more expensive, less effective, and less safe alternative antimicrobials [6]. In addition, drug-resistant bacteria are more likely to develop when broader-spectrum antimicrobials are used as alternatives. [6] Previous studies have reported a significant increase in surgical site infections due to the administration of other β-lactams as alternatives to perioperative antimicrobials in orthopaedic surgery during the cefazolin shortage [7,8]. Appropriate antimicrobial use is important for improving patient outcomes [9], and the antimicrobial stewardship team (AST) has an important role in responding to antimicrobial shortages [10,11].

Although there have been previous reports on MEPM shortages [10,12,13], one of these suggested a potential negative impact on in-hospital mortality [12]. Few studies have examined the impact of MEPM shortages on mortality in patients receiving antimicrobial therapy [13]. When responding to antimicrobial shortages, it is important to consider the potential negative impact of antimicrobial shortages on treatment. MEPM is a particularly reliable antimicrobial for severe infections and drug-resistant organisms, and the impact of MEPM shortages is of great concern. This retrospective study, conducted at a single centre in a medium-sized hospital in Japan, aimed to investigate the impact of MEPM shortages and responses on the use of broad-spectrum antimicrobials and treatment outcomes.

## Methods

### Study design and data source

This retrospective study was conducted at National Hospital Organization (NHO) Sagamihara Hospital in Japan, a 458-bed secondary medical care facility with four intensive care beds. Our hospital has no infectious disease department. The AST was organised and started work in April 2019. AST has no infectious disease physicians, but is staffed by three physicians without infectious disease certification, a dedicated clinical pharmacist, an infection prevention nurse and a clinical microbiologist in our hospital.

We retrospectively analysed the clinical data of patients who received the broad-spectrum antimicrobials in our hospital. The medical records of these patients were reviewed by three individuals from March 2022 to February 2023. Days of therapy (DOT) and days of antibiotic spectrum coverage (DASC) for broad-spectrum antimicrobials were calculated as indicators of antimicrobial use in patients receiving broad-spectrum antimicrobials. The period from March 2022 to August 2022 was defined as the MEPM pre-shortage period, and the period from September 2022 to February 2023 was defined as the MEPM shortage period. MEPM, tazobactam/piperacillin (TAZ/PIPC), cefepime (CFPM), levofloxacin (LVFX), and ciprofloxacin (CPFX) were collectively categorised as broad-spectrum antimicrobials.

### Antimicrobial stewardship program

Our hospitals do not use an antimicrobial preauthorization system, but use a reporting system for prescribing some intravenous broad-spectrum antimicrobials. The target antimicrobials in the reporting system were vancomycin, teicoplanin, daptomycin, linezolid and MEPM. In the reporting system, in order to prescribe these antimicrobials, the physician must enter patient information on a specific sheet, including the type of infection, the underlying disease, whether a culture has been submitted, and the planned duration of administration. This reporting system is managed by AST and provides instant access to patients and their information.

Since October 2021, prior to the MEPM shortage, AST had been performing daily PPRF on patients receiving intravenous broad-spectrum antimicrobials. The PPRF included patients receiving intravenous TAZ/PIPC, CFPM, LVFX and CPFX in addition to the reporting system antimicrobials. In the PPRF, an AST pharmacist has identified patients receiving broad-spectrum antimicrobials from the electronic medical record and performed the PPRF based on the type of infection and the presence or absence of microbiological tests and their results. Our hospital has performed drug susceptibility testing using the broth microdilution method according to the standards of the Clinical and Laboratory Standards Institute. We have detected drug-resistant bacteria such as methicillin-resistant *Staphylococcus aureus*, penicillin-resistant *Streptococcus pneumoniae*, extended-spectrum betalactamase (ESBL)-producing Enterobacterales (PE), AmpC betalactamase-PE and multidrug-resistant *Pseudomonas aeruginosa*. When AmpC betalactamase-PE were detected, if the minimum inhibitory concentrations (MICs) of CFPM were above 4 μg/mL, it was checked whether ESBLs were also produced. These patients were monitored for appropriate antimicrobial selection according to their concomitant medications and appropriate antimicrobial dosing according to their weight, liver and renal function. Treatment was monitored using vital signs, blood tests and imaging studies. Cases where these checks required a physician's decision were discussed with the AST-affiliated physicians. When the AST pharmacist needed to assist the physician, suggestions were made by the AST pharmacist either directly or through the ward pharmacist. In addition, as with PPRF, patients with positive blood cultures are monitored and feedback is provided daily from the time the blood culture is positive.

As an educational support, we conducted at least two workshops per year for hospital staff on the appropriate use of antimicrobials.

### Response to MEPM shortage

MEPM is the only carbapenem antimicrobial used in our hospital. MEPM was recommended for use in patients with severe infections such as sepsis, septic shock or infections caused by drug-resistant bacteria such as extended-spectrum or AmpC betalactamase-PE.

In August 2022, the supply of MEPM from generic MEPM manufacturer was discontinued and the hospital adopted MEPM from branded MEPM manufacturer. However, the potential supply of MEPM from the branded manufacturer was only about 30% of the actual consumption per month based on the previous year's consumption. Therefore, the AST and pharmacy departments disseminated information about the limited supply of MEPM to all departments. During the MEPM shortage, the AST pharmacist also reported the monthly use and supply of MEPM at antimicrobial stewardship meetings attended by representatives from each sector. There were no supply restrictions for intravenous antibiotics other than MEPM. Newer antimicrobials for multidrug-resistant gram-negative organisms (e.g. imipenem/cilastatin/relebactam, ceftazidime/avibactam and ceftolozane/tazobactam) were not included in the formulary at our hospital.

AST has also developed a list of alternatives to MEPM, with reference to antimicrobial spectrum and efficacy information. Physicians could access the list at any time in their electronic health record. In the list, for infections caused by ESBL-PE, cefmetazole was recommended as an alternative for bacteremic or non-bacteremic urinary tract infections (UTIs) and TAZ/PIPC for non-bacteremic UTIs based on previous studies [[14], [15], [16], [17]]. CFPM for infections caused by AmpC betalactamase-PE, with CFPM MICs of 2 μg/mL or less, and CFPM for febrile neutropenia based on previous studies [[18], [19], [20], [21], [22], [23]]. However, TAZ/PIPC was not recommended for infections caused by AmpC betalactamase-PE because of possible negative effects from previous studies [21,24,25]. Other broad-spectrum antimicrobials covering *Pseudomonas aeruginosa* on the list of alternatives are LVFX and CPFX.

The strategy to address MEPM shortages through the PPRF was not to restrict the initiation of MEPM use but to support physicians prescribing MEPM to switch to alternative antimicrobials or de-escalate to other antimicrobials depending on the type of infection or organism. For severe infections equivalent to sepsis or septic shock, de-escalation is recommended based on improvement in organ damage and withdrawal of vasopressors, depending on the organism. For example, first-line drugs were recommended for the causative organisms, such as ampicillin for *Streptococcus* spp. and *Enterococcus faecalis*, and cefazolin for methicillin-susceptible *Staphylococcus aureus*, *Escherichia coli* and *Klebsiella pneumoniae*.

In March 2023, the MEPM supply of generic manufacturer was resumed and reused from that date.

## Outcomes

### DOT and DASC of broad-spectrum antimicrobials

DOT and DASC of broad-spectrum antimicrobials before and during the MEPM shortage were compared. DOT was calculated per 1000 patient-days and every two weeks. It has been suggested that DASC is an effective metric for evaluating antimicrobial stewardship efforts compared to DOT [26,27]. DASC was computed using the antibiotic spectrum coverage (ASC) score, as defined in a previous study, together with the number of days of administration [26]. DASC was calculated per 1000 patient-days and assessed every two weeks.

### Number of cases and median days of MEPM administration

In patients receiving MEPM, we compared the number of cases per 1000 admissions and the median days of administration before and during the MEPM shortage. These values were calculated every two weeks, based on the start date of MEPM administration.

### Thirty-day in-hospital mortality rates for patients receiving broad-spectrum antimicrobials

To assess the impact of the MEPM shortage on the treatment of severe infections, we compared the 30-day in-hospital mortality rate of patients treated with broad-spectrum antimicrobials before and during the MEPM shortage. The mortality rate, calculated every two weeks, is the number of deaths per 1,000 admissions. Deaths were defined as in-hospital deaths within 30 days of initiating broad-spectrum antimicrobial therapy. Patients who died on the day of admission and those who received only one day of broad-spectrum antimicrobial therapy were excluded from the study.

## Statistical analysis

In this study, we conducted an interrupted time-series analysis (ITSA) to assess the impact of the MEPM shortage on outcomes. Thirteen data points were included for both the pre-shortage and shortage periods, resulting in a total of twenty-six data points for the ITSA. The model included an intercept, baseline trend, level change, and trend change after the onset of the MEPM shortage. Statistical analysis was performed using STATA version 18.0 (StataCorp LP, College Station, TX), with all significance levels set at p < 0.05.

## Ethics approval

This study was conducted in accordance with the Ethical Guidelines for Medical Research Involving Human Subjects, with the utmost care for the protection of personal information. The study was approved by the Ethical Review Committee of our institution (approval number: NHO National Sagamihara Hospital 2023-003). The study adhered to the tenets of the Declaration of Helsinki.

## Results

### DOT and DASC of broad-spectrum antimicrobials

**Figure 1:**
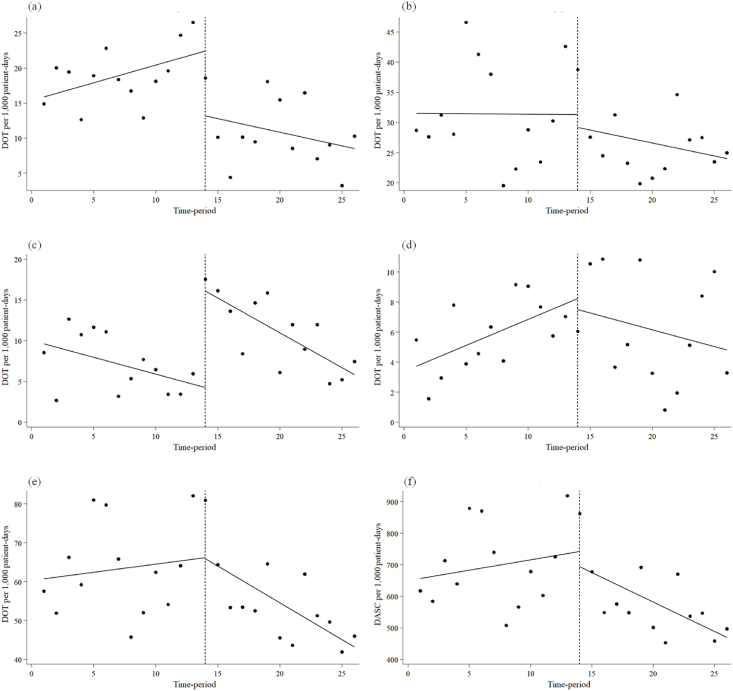
Interrupted time-series analysis of DOT and DASC (per 1,000 patient-days) of broad-spectrum antimicrobials before and during the meropenem shortage period. (a) Meropenem, (b) Tazobactam/piperacillin, (c) Cefepime, (d) Fluoroquinolones, (e) Overall broad-spectrum antimicrobials, (f) Overall broad-spectrum antimicrobials.

Figure 1 and Table I present the DOT and DASC of broad-spectrum antimicrobials before and during the MEPM shortage period. MEPM DOT exhibited no significant slope before the shortage period (0.51; 95% confidence interval [CI]: -0.11 to 1.12, p = 0.10). However, there was an immediate decrease during the shortage period (-9.28; 95% CI: -16.99 to -1.57, p < 0.05), with no subsequent decreasing trend (-0.89; 95% CI: -1.90 to 0.12, p = 0.08). CFPM DOT showed no significant slope before the shortage period (-0.41; 95% CI: -0.90 to 0.08, p = 0.09). An immediate increase was observed during the shortage period (11.84; 95% CI: 8.19 to 15.49, p < 0.001), but no upward trend followed (-0.44; 95% CI: -1.04 to 0.15, p = 0.14). Fluoroquinolone DOT exhibited a tendency to increase before the shortage period (0.35; 95% CI: 0.07 to 0.63, p < 0.05). However, no significant change in level occurred during the shortage period (-0.71; 95% CI: -4.69 to 3.27, p = 0.71), and there was no significant decreasing trend (-0.57; 95% CI: -1.17 to 0.02, p = 0.06). TAZ/PIPC DOT and overall broad-spectrum antimicrobials DOT and DASC for overall broad-spectrum antimicrobials showed no significant changes in slope before the shortage period, and no significant changes in levels and trends during the shortage period.

**Table I:** Interrupted time-series analysis of the impact of the meropenem shortage on the use of broad-spectrum antimicrobials

Parameter	Coefficient	95% CI	p-value
DOT of MEPM			
Baseline trend	0.51	-0.11, 1.12	0.10
Level change after the start of the MEPM shortage	-9.28	-16.99, -1.57	< 0.05
Trend change after the start of the MEPM shortage	-0.89	-1.90, 0.12	0.08
DOT of TAZ/PIPC			
Baseline trend	-0.01	-1.17, 1.14	0.98
Level change after the start of the MEPM shortage	-2.12	-12.86, 8.62	0.69
Trend change after the start of the MEPM shortage	-0.42	-2.00, 1.17	0.59
DOT of CFPM			
Baseline trend	-0.41	-0.90, 0.08	0.09
Level change after the start of the MEPM shortage	11.84	8.19, 15.49	< 0.001
Trend change after the start of the MEPM shortage	-0.44	-1.04, 0.15	0.14
DOT of fluoroquinolones			
Baseline trend	0.35	0.07, 0.63	< 0.05
Level change after the start of the MEPM shortage	-0.71	-4.69, 3.27	0.71
Trend change after the start of the MEPM shortage	-0.57	-1.17, 0.02	0.06
DOT of overall broad-spectrum antimicrobials			
Baseline trend	0.42	-1.22, 2.07	0.60
Level change after the start of the MEPM shortage	-0.27	-16.45, 15.90	0.97
Trend change after the start of the MEPM shortage	-2.32	-4.74, 0.10	0.06
DASC of overall broad-spectrum antimicrobials			
Baseline trend	6.59	-11.65, 24.83	0.46
Level change after the start of the MEPM shortage	-47.44	-230.87, 135.99	0.60
Trend change after the start of the MEPM shortage	-25.29	-52.24, 1.66	0.06

MEPM: meropenem; CI: confidence interval; DOT: days of therapy; TAZ/PIPC: tazobactam/piperacillin; CFPM: cefepime; Fluoroquinolones: levofloxacin, ciprofloxacin; Overall broad-spectrum antimicrobials: meropenem, tazobactam/piperacillin, cefepime, levofloxacin, ciprofloxacin; DASC: days of antibiotic spectrum coverage.

### Number of cases and median days of MEPM administration

**Figure 2:**
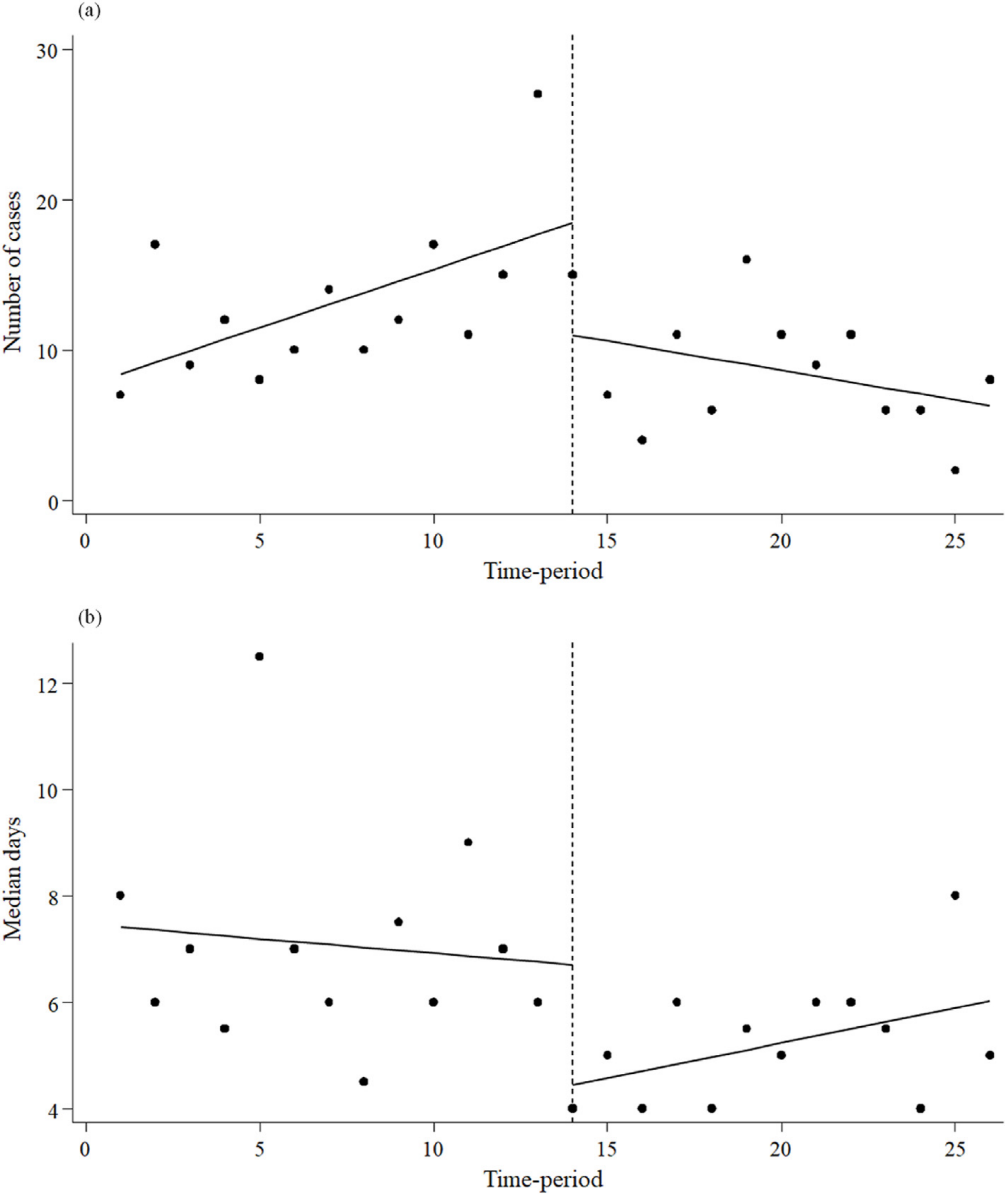
Interrupted time-series analysis of the number of cases and median days of meropenem administration before and during the meropenem shortage period. (a) Number of cases, (b) Median days.

Figure 2 and Table II illustrate the number of cases and median days of MEPM administration before and during the MEPM shortage period. The number of MEPM cases administered did not exhibit a significant slope before the shortage period (0.77; 95% CI: -0.05 to 1.60, p = 0.07). However, there was an immediate decrease during the shortage period (-7.47; 95% CI: -14.89 to -0.05, p < 0.05), and a decreasing trend was observed (-1.16; 95% CI: -2.27 to -0.06, p < 0.05). The median days of MEPM administration did not show a significant slope before the shortage period (-0.05 95% CI: -0.24 to 0.13, p = 0.54). However, an immediate decrease occurred during the shortage period (-2.25; 95% CI: -3.64 to -0.86, p < 0.05), with no subsequent decreasing trend (0.19; 95% CI: -0.03 to 0.40, p = 0.09).

**Table II:** Interrupted time-series analysis of the impact of the meropenem shortage on the number of cases and days of meropenem administration

Parameter	Coefficient	95% CI	p-value
Number of cases of MEPM administration			
Baseline trend	0.77	-0.05, 1.60	0.07
Level change after the start of the MEPM shortage	-7.47	-14.89, -0.05	< 0.05
Trend change after the start of the MEPM shortage	-1.16	-2.27, -0.06	< 0.05
Median days of MEPM administration			
Baseline trend	-0.05	-0.24, 0.13	0.54
Level change after the start of the MEPM shortage	-2.25	-3.64, -0.86	< 0.05
Trend change after the start of the MEPM shortage	0.19	-0.03, 0.40	0.09

MEPM: meropenem; CI: confidence interval.

During the MEPM shortage period, 32.4% of MEPM-treated patients received MEPM for more than seven days, details of which are given below. The types of infection were urinary tract infection (22.2%), respiratory tract infection and intra-abdominal infection (16.7%). In contrast, 52.8% of the attributable bacteria were unknown, 16.7% were ESBL-PE, and 5.6% were AmpC betalactamase-PE.

### Thirty-day in-hospital mortality rates for patients receiving broad-spectrum antimicrobials

**Figure 3:**
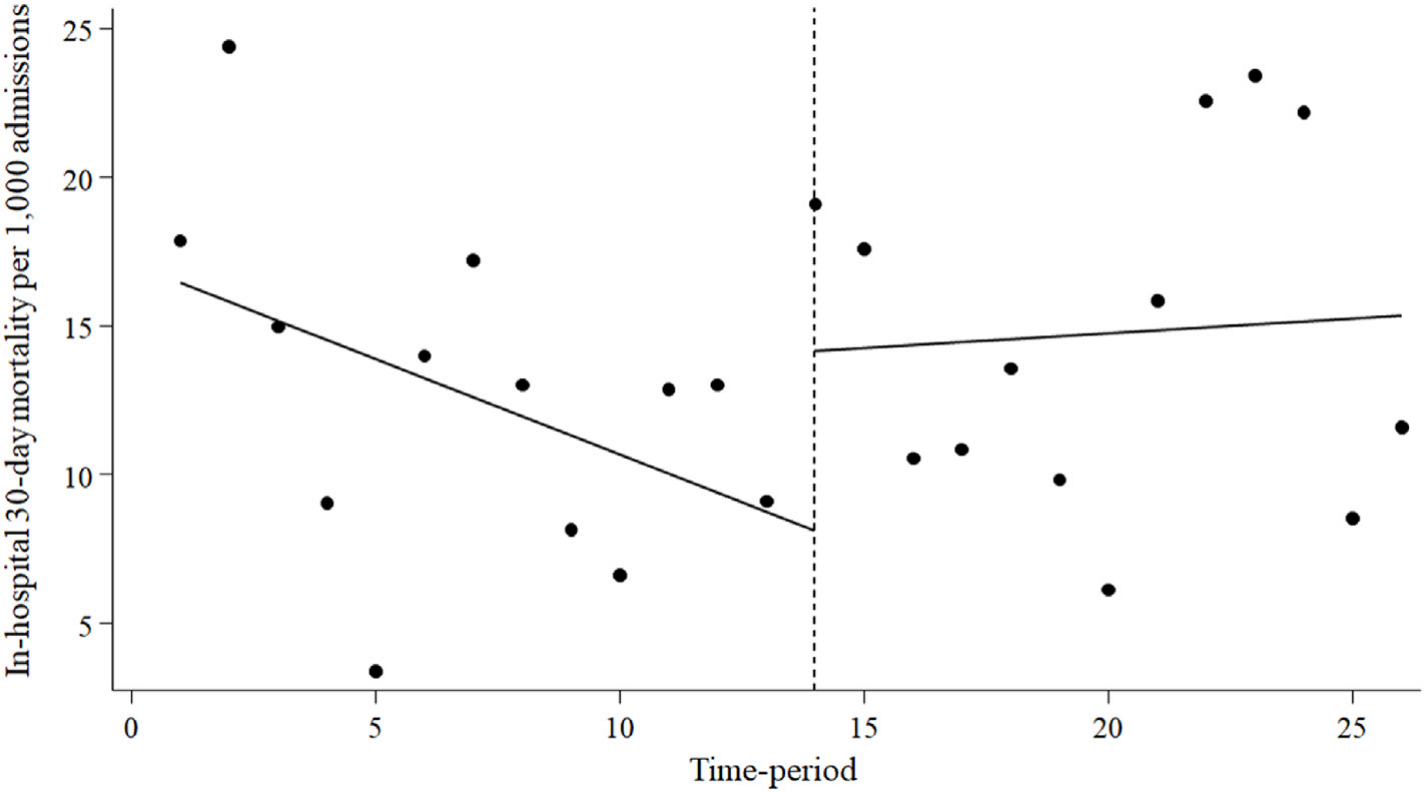
Interrupted time-series analysis of 30-day in-hospital mortality in patients using broad-spectrum antimicrobials per 1,000 admissions before and during the meropenem shortage period.

Figure 3 and Table III depict the 30-day in-hospital mortality rates for patients receiving broad-spectrum antimicrobials before and during the MEPM shortage period. The 30-day in-hospital mortality rate for these patients did not exhibit a significant slope before the MEPM shortage period (-0.64; 95% CI: -1.44 to 0.16, p = 0.11). Additionally, there were no significant changes in level (6.05; 95% CI: -1.32 to 13.42, p = 0.10) or trend (0.74; 95% CI: -0.52 to 2.00, p = 0.24) during the shortage period.

**Table III:** Interrupted time-series analysis of the impact of the meropenem shortage on 30-day in-hospital mortality in patients using broad-spectrum antimicrobials

Parameter	Coefficient	95% CI	p-value
In-hospital 30-day mortality per 1,000 admissions in patients using broad-spectrum antimicrobials			
Baseline trend	-0.64	-1.44, 0.16	0.11
Level change after the start of the MEPM shortage	6.05	-1.32, 13.42	0.10
Trend change after the start of the MEPM shortage	0.74	-0.52, 2.00	0.24

MEPM: meropenem; CI: confidence interval; DOT: days of therapy; broad-spectrum antimicrobials: Meropenem, Tazobactam/Piperacillin, Cefepime, Levofloxacin, Ciprofloxacin.

## Discussion

We employed ITSA, a robust quasi-experimental method, to assess the influence of the MEPM shortage and PPRF on the utilisation and treatment outcomes of broad-spectrum antimicrobials, encompassing MEPM. The results of this study showed a decrease in MEPM DOT and an increase in CFPM DOT immediately following the MEPM shortage. Subsequently, there was a sustained decrease in DOT for MEPM, while a decrease in DOT for broad-spectrum antimicrobials, including CFPM, was suggested. Crucially, there were no immediate or sustained alterations in 30-day in-hospital mortality rates among patients receiving broad-spectrum antimicrobials during the MEPM shortage period.

The use of MEPM in our hospital was low before the study period. The period before the MEPM shortage was the first time that daily PPRF was used, and AST recommended the use of MEPM for severe infections and infections caused by drug-resistant bacteria. This recommendation may have also contributed to a lower threshold for physician use of MEPM, leading to an increase in the number of patients using MEPM (Figure 2) and an increase in the DOT for MEPM (Figure 1). However, for patients who did not require MEPM, the AST have recommended switching to other antimicrobials through the PPRF, resulting in fewer days of treatment (Figure 2). In contrast, Figure 2 and Table II indicate that the decrease in MEPM DOT during the MEPM shortage was influenced more by the reduction in the number of cases administered with MEPM than by the decrease in the number of days administered with MEPM. The decline in the number of MEPM cases administered immediately after the MEPM shortage is assumed to be due to the news of the MEPM shortage. A sustained downward trend in the number of cases administered with MEPM was likely due to the careful selection of cases administered with MEPM, driven by the growing recognition of the shortage and the implementation of PPRF. Previous studies on PPRF for carbapenems have reported a decrease in carbapenem use due to increased de-escalation and reduced days of administration [28]. It is suggested that the present study did not observe a reduction in the number of days of administration during the MEPM shortage period because PPRF had already been performed before the MEPM shortage period. The trend towards a longer duration of MEPM administration as the MEPM shortage continued, although not statistically significant, is thought to be due to the careful selection of cases administered MEPM, with the proportion of cases requiring administration increasing even after the results of various cultures were known. In fact, cases in which MEPM was administered for more than seven days often included severe infections with unknown causative organisms and intra-abdominal and respiratory infections caused by ESBL-PE. In the former cases, MEPM was considered to be continuously administered because the cases were hospital-acquired or switched from other antimicrobials due to poor efficacy, and the involvement of resistant bacteria could not be ruled out. In the latter cases, the patient was not switched to another antimicrobial, possibly because of non-UTIs caused by ESBL-PE.

On the other hand, the immediate increase in the DOT for CFPM during the shortage period could be due to its overuse, as it is often included in alternative drug lists. The subsequent downward trend in CFPM DOT may be due to the reduction in unnecessary use of CFPM due to our PPRF for patients receiving CFPM. Drawing from findings in earlier studies, it is logical to anticipate the utilisation of other broad-spectrum antimicrobials as substitutes, with increased usage during periods of shortages in broad-spectrum antimicrobials. Reports have indicated heightened use of TAZ/PIPC and CFPM during MEPM shortages [12]. Other studies noted increased usage of MEPM or CFPM during TAZ/PIPC shortages [11,29,30], and a rise in the use of MEPM and TAZ/PIPC during the CFPM shortage [31]. There have also been reports of increased use of TAZ/PIPC and CFPM, instead of decreasing the use of carbapenems, due to antimicrobial stewardship (AS) [32,33]. It should be noted that if the use of broad-spectrum antimicrobials decreases, other broad-spectrum antimicrobials may be used instead and their use may increase. However, during the MEPM shortage period in this study, the trend for broad-spectrum antimicrobial DOT and DASC did not change statistically significantly, but visually there was a clear decrease over time (Figure 1). The decrease in these indicators predicts that non-broad-spectrum antimicrobials may have been used in addition to broad-spectrum antimicrobials to preserve MEPM during the shortage period. It is suggested that the MEPM shortage and the PPRF initiative in our hospital may have increased awareness of the appropriate use of antimicrobials for physicians, including avoiding unnecessary use of broad-spectrum antimicrobials, taking into account the type of infection and causative organisms. PPRF has also been suggested to have an educational and awareness-raising effect on the appropriate use of antimicrobials [34], and our PPRF has also shown an important role in reducing the inappropriate use of broad-spectrum antimicrobials, an alternative to MEPM.

Up to the present, there have been no reported instances of MEPM shortages with discernible negative treatment effects. However, this is not a negative effect that can be ruled out, as it is unlikely to have been studied and negative effects are less likely to be reported [35]. In this study, the mortality outcomes for patients undergoing broad-spectrum antimicrobial treatment indicate that the MEPM shortage and PPRF initiatives did not adversely impact treatment. We suspect that our approach of not restricting the initiation of MEPM, but rather supporting the change or discontinuation of treatment through daily PPRF for MEPM recipients, may have contributed to this. We also drew on previous studies to develop a list of alternatives to MEPM, supporting the selection of appropriate antimicrobials for causative microorganisms and infections. In cases of severe infections, improper initial drug selection is a key factor in heightened mortality [36,37]. To preserve carbapenems, alternatives for drug-resistant bacteria are being actively researched. Recent reports also suggest that CMZ may be an alternative to carbapenems for ESBL-PE bacteraemia [38]. The use of CFPM, rather than TAZ/PIPC, for infections caused by AmpC betalactamase-PE is recommended in other reports [39,40]. However, CFPM is not recommended as an alternative to carbapenems for infections caused by ESBL and AmpC betalactamase co-producing Enterobacterales [40]. Therefore, depending on the type of infection and the causative organism, there are different antimicrobial alternatives to MEPM that are effective and safe. In other words, a AS-based carbapenem conservative strategy requires the use of the most appropriate antimicrobials for each patient, while keeping the information up to date.

This study had several limitations. Firstly, as a retrospective observational study conducted within a single institution, extrapolating findings to other institutions poses challenges. Variables such as institution size, characteristics, patterns of antimicrobial usage and adoption, alongside the extent of supply restrictions, collectively influence antimicrobial utilisation. Secondly, the study relied on relatively short-term data due to the time constraints imposed by the period between supply restrictions and the restoration of supply. ITSA enhances the ability to identify intervention effects as the number of data points increases, fostering higher correlations [41,42]. Prolonged supply restrictions beyond the scope of this study could potentially lead to severe MEPM shortages, resulting in more profound usage limitations and potentially distinct impacts. Unfortunately, the impact on the incidence of carbapenem-resistant and multidrug-resistant *Pseudomonas aeruginosa* could not be evaluated due to the abbreviated duration of the MEPM shortage. While reports on bacterial resistance due to antimicrobial supply restrictions are limited, studies on CFPM supply constraints suggest a decline in susceptibility rates of *Pseudomonas aeruginosa* with the escalated use of alternative agents [29]. Subsequent studies should delve into the long-term impact of MEPM shortages on severe infection treatment and drug susceptibility.

## Conclusions

The results of this study state that soon after the shortage of MEPM, the use of MEPM decreased and the use of CFPM as an alternative temporarily increased. Subsequently, despite a continued decline in the use of MEPM, there was some indication of a decline in the use of broad-spectrum antimicrobials, including CFPM. The shortage of MEPM has led physicians to administer alternative antimicrobials, including broad-spectrum antimicrobials, with greater consideration of the severity of the infection and the causative organisms. AST should also support physicians in ensuring that evidence-based antimicrobials, including alternatives to MEPM, are administered for successful MEPM conservation strategies.

Most importantly, the results suggest that there were no adverse effects on mortality in patients receiving broad-spectrum antimicrobials, which had been a concern. The application of ITSA facilitated the visualisation of both immediate and enduring effects of the MEPM shortage. Antimicrobial shortages pose a significant obstacle to the administration of necessary treatments. However, conversely, such shortages might offer a valuable opportunity to reassess the judicious use of antimicrobials in each hospital.

## Credit author statement

Kohei Maruyama: Conceptualization; Formal analysis; Investigation; Project administration; Visualization; Writing - original draft.

Kiyoshi Sekiya: Formal analysis; Project administration; Supervision.

Noriyuki Yanagida: Formal analysis; Investigation; Writing - review & editing.

Shuhei Yasuda: Data curation; Visualization.

Daisuke Fukumoto: Methodology; Project administration.

Satoshi Hosoya: Data curation; Methodology; Writing - review & editing.

Hiromitsu Moriya: Data curation; Methodology; Visualization.

Motoko Kawabe: Formal analysis; Investigation; Project administration.

Tatsuya Mori: Conceptualization; Supervision; Writing - review & editing.

## Acknowledgments

None.

## Conflict of interest statement

None to declare.

## Funding statement

This research did not receive any specific grant from funding agencies in the public, commercial, or not-for-profit sectors.
